# Structured Porous Material Design for Passive Flow and Noise Control of Cylinders in Uniform Flow

**DOI:** 10.3390/ma12182905

**Published:** 2019-09-09

**Authors:** Elias J. G. Arcondoulis, Yu Liu, Zhiyong Li, Yannian Yang, Yong Wang

**Affiliations:** 1 Department of Mechanics and Aerospace Engineering, Southern University of Science and Technology, Shenzhen 518055, China (E.J.G.A.) (Z.L.) (Y.Y.); 2 Key Laboratory of Aerodynamic Noise Control, China Aerodynamics Research and Development Center, Mianyang 621000, China

**Keywords:** porous material, aeroacoustics, passive noise control

## Abstract

Cylindrical bodies in uniform flows can be coated with a porous medium as a passive flow and noise control method in an effort to reduce the acoustic effects of vortex shedding. To date, the employed open-cell porous materials typically possess a randomized internal structure. This paper presents the design and validation of a novel 3-D printed structured porous coated cylinder that has significant flexibility, in that the porosity and pores per inch of the porous coating can be modified independently and relatively easily. The performance of the structured porous coating design is compared against porous polyurethane and metal foam with the same coating dimensions and similar pores per inch and porosity via an experimental acoustic investigation, revealing strong similarity in the passive noise control performance of each material type. A numerical comparison illustrates the similarities of the wake structure of the 3-D printed porous coated cylinder to an equivalent Darcy–Forchheimer model simulation that represents a randomized internal porous structure. The performance similarities of these different porous material types indicate that a structured porous geometry can be used to understand the internal flow behavior of the porous medium responsible for reducing the cylinder vortex shedding tone that is otherwise extremely difficult or impossible with typical randomized porous structures. Moreover, significant potential exists for the porous structure to be further optimized or smartly tailored by architectural design for different control purposes, coating geometries and dimensions, and working conditions.

## 1. Introduction

Passive flow control methods have become increasingly important and popular in commercial and industrial applications that display bluff body aeroacoustic noise. Porous coatings of bluff bodies is a relatively new method of passive flow control. The investigation of porous coated cylinders is of specific interest, as the cylinder can pose as a simplistic representation of typical noisy engineering components due to aeroacoustic interaction, such as an aircraft landing gear [1] and parts of high speed train pantographs [2]. Vortex shedding control of a cylinder was investigated by Zradkovich [3] who applied a metal foam coating to a cylinder surface to minimize the effects of vortex shedding and claimed that a prolonged wake could minimize the lift and drag force fluctuations acting on the cylinder. Sueki et al. [4] demonstrated using Particle Image Velocimetry (PIV) that the wake of the porous coated cylinder is predominantly zero velocity and Aguiar et al. [5] observed using Laser Doppler Anemometry a more stabilized wake and a significant reduction in pressure drag coefficient. Ruck et al. [6] and Klausmann and Ruck [7] experimentally investigated the effects of replacing a segment of the leeward side of a cylinder with a porous medium, revealing significant drag reduction for all material porosities tested. Geyer et al. [8] observed that by applying a porous coating to a cylinder did not suppress the vortex shedding tone but widened its spectral peak for a range of porous materials and porosities. This research was further investigated, revealing that the porous coating of a cylinder actually in some flow conditions strengthened the vortex shedding tone but decreased much of the broadband noise [9]. Their more recent numerical simulations [10] agree with their experimental data. For comparative purposes, it should be noted that they used far less porous materials than the aforementioned literature. Furthermore, porous coated tandem cylinder configurations have displayed significant overall noise reduction [11,12]. Both the tonal frequency and magnitude were shown to decrease via the application of a porous coating, which is consistent with the numerical findings of Liu et al. [13] who analyzed a single porous coated cylinder.

**Figure 1:**
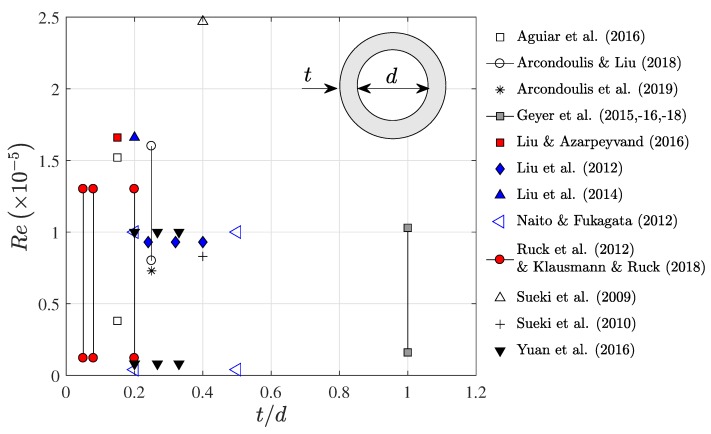
Collation of numerical and experimental porous coated cylinder studies. Lines have been drawn between points where experiments have been conducted at many Reynolds numbers for a specific cylinder configuration. The quantities *t* and *d* are labeled on a schematic diagram of a porous coated cylinder.

Figure 1 displays a collation of data of porous coated cylinder studies, where it can be observed that the majority of studies employ cylinders with a porous cover thickness to bare cylinder diameter ratio, t/d, of 0.1 to 0.5 and within Reynolds numbers, Re=UD/ν = $0.1\times 10^{5}$ to $1.8\times 10^{5}$, where *U* is the freestream flow speed, *D* is the cylinder outer diameter and ν is the kinematic viscosity of the fluid.

By collation of the data within Figure 1 average quantities are calculated; t/d≈0.22, average minimum Reynolds number $Re_{\mathrm{av},\min}\approx 0.8\times 10^{5}$ and an average maximum Reynolds number $Re_{\mathrm{av},\max}\approx 1.5\times 10^{5}$. The important properties of porous media include the numbers of Pores Per Inch (PPI) and the porosity, ϕ (as a percentage of the porous media volume with respect to a solid equivalent). The typical experimentally investigated PPI range, using metal foam and polyurethane, is from 10 PPI to 30 PPI [2,4,5,6,14], with significant vortex shedding tone reduction observed using 10 PPI materials [5,6,14]. It should be noted that Sueki et al. [2] experimentally showed that the material type (i.e., comparing metal foam to polyurethane) had little or no impact on the measured noise reduction and therefore data for different material types (if they possess the same PPI and porosity) can be compared fairly.

There are many flow characteristics of bare, non-treated cylinders that are understood at length [15,16], yet this is not the case with porous coated cylinders. It is currently extremely difficult to provide deeper insight into the internal flow structure of randomized porous media, both experimentally and numerically (without using approximation methods), due to the complexity of the internal structure and the lack of a consistent clear Line-Of-Sight (LOS) from the outer to the inner diameter. These difficulties and others have led to very few publications regarding the fundamental fluid mechanics properties of porous coated cylinders. Bruneau and Motazavi [17] developed a numerical method to simulate fluid flows within porous regions, which they used to show a significant decrease in vortex induced vibrations for several industrial and commercial applications. At low Reynolds numbers, Xu et al. [18] conducted a numerical investigation of 3-D flow through a single pore of open-cell metal foam, revealing the internal flow field within a single pore; yet, it is difficult to determine the influence of the cylindrical geometry on the internal flow field as compared to a single, isolated pore. Despite these numerical simulations, there are no experimental data to verify these results. Understanding important fundamental flow characteristics, such as spanwise coherence length [12], development of the boundary layer within the porous structure [19,20] and regions of instabilities within the cylinder wake [21] will help enable the design of improved or optimized porous materials not only for cylinders, but for the bluff bodies that they represent [1,2,4].

One option is to use an Index Refraction Matching method [22] whereby a transparent body is tested in a water tunnel facility. By adding saline-based chemicals into the water, the refractive indices of the material body and the fluid can be matched, thereby making the body totally transparent. Then by using planar-PIV or tomographic PIV of a transparent cylinder [20], the internal flow field of the porous coated cylinder can be recorded. While this method is attractive, the material used for the porous coated cylinder must be highly transparent and therefore either CNC-machined or 3-D printed, thereby eliminating the use of readily available materials possessing a randomized internal porous structure.

This paper presents the design and validation of a customizable structured porous material, applied as a coating to cylinder subject to uniform flow. This structured porous coated cylinder possesses similar passive flow and noise control properties to the tested randomized porous materials, being metal foam and porous polyurethane. The consequence of these similarities is that the structured porous material can be used to understand the fundamental fluid mechanics of other randomized porous media due to the relative simplicity of its design. The structured porous material possesses clear LOS in both the radial and spanwise directions, allowing the implementation of more advanced flow measurement techniques to investigate the internal flow field. Furthermore, the internal structure is dictated by a single c-shaped component that allows the control of and independence between the material PPI and porosity.

## 2. Materials and Methods

### 2.1. Porous Materials

**Figure 2:**
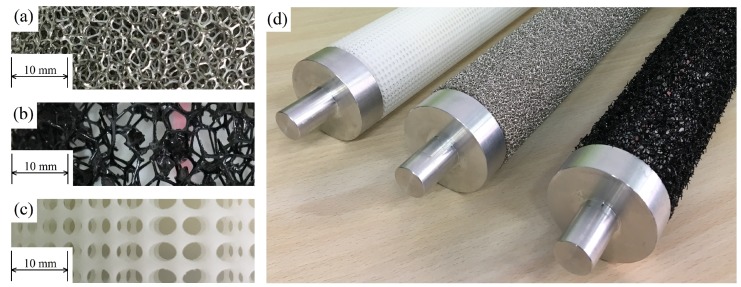
Photos of the PCC materials used in this study, applied to a cylindrical body. (**a**) Nickel-metal foam obtained from Kunshan Tengerhui Electronic Technology Co., Ltd., (**b**) Porous polyurethane obtained from Shenzhen Shiyuan Foam Co., Ltd. and (**c**) Structured porous coating manufactured via 3-D printing. Image (**d**) shows the three PCCs with their wind tunnel fittings attached. All materials were manufactured in Shenzhen, China.

Three types of Porous Coated Cylinders (PCCs) are investigated in this study: a Structured Porous Coated Cylinder (SPCC) and for comparative purposes, a Metal Foam Porous Coated Cylinder (MFPCC) and a Polyurethane Porous Coated Cylinder (PUPCC). Photographs of the PCCs used in these experiments are presented in Figure 2, including enlargements of the porous coatings to help visualize the difference between the structured and randomized pore structures. Details of the SPCC design and manufacture are provided in the subsequent sections.

### 2.2. External Design

An iterative design process of many interacting factors is investigated during the PCC design phase, such as the Reynolds number, outer diameter *D* and thickness-to-bare-diameter ratio t/d, expected shedding tone frequency *f* and manufacturing limitations. Based on the typical background noise characteristics of anechoic wind tunnel facilities [14,23], it is desired to use as small an outer-diameter cylinder as possible to increase the frequency of the vortex shedding frequency tone that is produced in uniform flow to surpass the anechoic cut-off frequency of the facility. The Strouhal number, St=fD/U, was used to determine the expected shedding tone frequency of a porous coated cylinder. It was assumed that both the bare and porous cylinders will exhibit an approximate Strouhal number of 0.18 [16] within the Reynolds number range adopted in this study.

The Reynolds number range of interest is based on published data of both porous coated [2,4,5,6,8,12,13,19,24] and bare cylinders [16,25,26,27] corresponding to $Re_{\mathrm{av},\min}\approx 0.8\times 10^{5}$ to $Re_{\mathrm{av},\max}\approx 1.5\times 10^{5}$. Therefore a value of *D* is required such that a similar Reynolds number range could be obtained while also producing shedding tones that are above the anechoic limits of the wind tunnel (approximately 200 Hz) and well-exceed the background noise (that typically decreases with increasing frequency). Other than the shedding tone frequency, a smaller diameter is desired for several reasons, being an expected cheaper manufacturing cost (less total material) and a smaller blockage ratio of the wind tunnel test section. Based on an extensive search of 3-D printing manufacturers, the minimum cylinder diameter of a porous coated cylinder design that can be 3-D printed is restricted by the minimum thickness of any member within the porous structure. In order to use a smaller diameter cylinder, the relative size of the pores must increase to ensure that the PPI of cylinder is the same or at least close to the MFPCC and PUPPC. By increasing the pore size for a fixed diameter cylinder, this leads to thinner structural members within the ring shape (and subsequently much higher porosity) which thus makes the 3-D printing process either much more complicated, expensive or simply impossible. After extensive trial-and-error efforts, the PCC outer diameter was chosen as *D* = 60 mm.

By collating the data of previous research as presented in Section 1, the average t/d ratio is approximately 0.22. Therefore, for simplicity of design and manufacture (especially for the acquisition of a specific bare cylinder outer diameter from local suppliers), the PCCs used in this study possess t/d = 0.25 to provide comparable data. As D=d+2t, the porous coating has thickness, *t* = 10 mm and the bare cylinder has a diameter, *d* = 40 mm. Each PCC has a span of *L* = 360 mm.

### 2.3. Internal Design

The PCC’s PPI was dictated by local supplier availability and ensuring that the metal foam could be successfully bent from a sheet into a cylindrical shape without fracture of the open-cell porous structure or non-circular deformity (i.e., causing the coating to produce an elliptical shape). PCCs possessing 10 PPI have been shown to substantially reduce the cylindrical vortex shedding tone [5,6] as discussed in Section 1 and as a convenient consequence, this material was bent around a solid cylinder and the ends were sealed using a weld. In addition, 10 PPI porous polyurethane is readily available, thus making a selection of 10 PPI convenient and thereby a design parameter for the SPCC.

**Figure 3:**
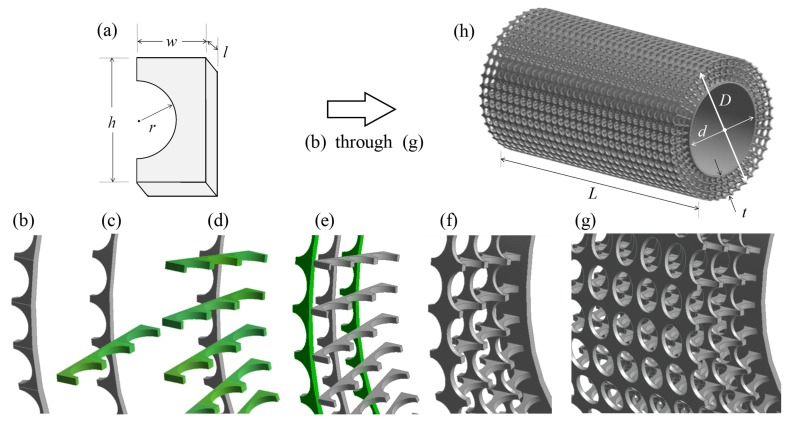
Stages of the creation of the SPCC using the ANSYS Workbench Geometry CAD package. Image (**a**) depicts the c-shaped chip piece that dictates the overall PPI and porosity of the SPCC. Stages (**b**) through (**e**) illustrate the components required to create the SPCC, where the addition from the previous step is illuminated in a green shading. Image (**f**) represents a mirrored-duplication of (**e**) about the vertical axis and (**g**) several duplications of (**f**) along the spanwise direction to produce image (**h**) the final SPCC design, labeled with key dimensions.

The SPCC design consists of a series of concentric interconnected c-shaped pieces, identified in Figure 3a. The motivation to use such a small piece and then replicate it many times was driven by the difficulty of 3-D printing straight rectangular members, as they become unstable under their own weight during printing. Circular members, analogous to the construction of an arch geometry, avoid these structural issues for the same structural member thickness by reducing the effects of bending moment stresses by redistributing the load via hoop stresses around the circular arc.

The dimensions *w*, *h*, *r* and *l* determine the SPCC overall PPI and porosity. Adjusting *w* influences the material porosity but not the PPI in the spanwise direction. Increasing *r* leads to increased porosity yet it does not influence the overall PPI. By ensuring *h* and *r* remain independent and h>r, *h* can be used as a parameter to influence the PPI in the circumferential direction and the porosity. Changing *w* influences the PPI in the spanwise direction and the porosity. The parameter *l* determines the thickness of each of the structural members of the porous material, thus influencing the porosity only. For the SPCC presented in this paper, the parameter *l* is dictated by a minimum allowable structural member thickness of a 3-D printed model.

Via manipulation of the parameters presented in Figure 3a, it can be seen that control of the porous material PPI and porosity can be obtained. It is recommended during the initial design stages to keep some of the parameters fixed, prior to varying some parameters to arrive at a desired SPCC. During the design process of the SPCC in this paper, the PPI of the SPCC was modified by adjusting *r* and *h* after establishing suitable values of *w* and *l* to ensure that the same (or very similar) PPI as the MFPCC and PUPCC was achieved. For the SPCC investigated in this paper, *r* = 1.1 mm, *h* = 3.4 mm, *w* = 1.75 mm and *l* = 1 mm. The reasoning for these specific parameters is to achieve 10 PPI and a porosity comparable to the metal foam and porous polyurethane (the evaluation of the PPI and porosity of the SPCC is presented after explaining the SPCC design within Section 2.3).

Each of the c-shaped pieces in Figure 3a are merged in a radial pattern by using an identical c-shaped piece at 90${}^{\circ }$, arranged circumferentially around the cylinder diameter as depicted in Figure 3b. The merging causes a non-smooth interface between the two pieces, forming a series of equispaced ridges. Circular cutting extrusions are applied to the upper and lower surfaces, such that the ridges are removed forming a smooth circular ring. The following step involves using three-connected c-shapes oriented in the opposite direction and mating with the ring thickness (i.e., the surface created by the area h×l), as illustrated by the green shading in Figure 3c. The thickness *l* of Figure 3b aligns with the h-2r parts of the three-connected c-shapes. The three-connected c-shapes are then cloned in an equispaced-pattern around the smooth circular ring as shown in Figure 3d to produce the overall porous coating thickness *t* and therefore also *D*. The smooth circular ring of Figure 3b is then cloned and scaled twice; the largest ring corresponding to the cylinder outer diameter *D* and the other mid-way between the original ring and the new outer diameter. The scaling only occurs in the radial direction, not in the spanwise direction. Therefore, the pores at the inner diameter interface are circular and grow elliptically in the radial direction. By using these scaling factors, the soon-to-be-pores are aligned on both axes, as depicted in Figure 3e. The geometry of Figure 3e is then mirrored about its principal axis. A thin annulus is created to produce the cylinder inner diameter, *d*, thus producing the SPCC blueprint shown in Figure 3f. Please note that *d* corresponds to the outer diameter of this thin annulus and that the inner diameter of the annulus is arbitrary. Each of these blueprints can be cloned in the spanwise direction with a smooth connecting interface between each of the porous structural members, as depicted in Figure 3g, producing the final SPCC design in Figure 3h of a desired span of *L*.

**Figure 4:**
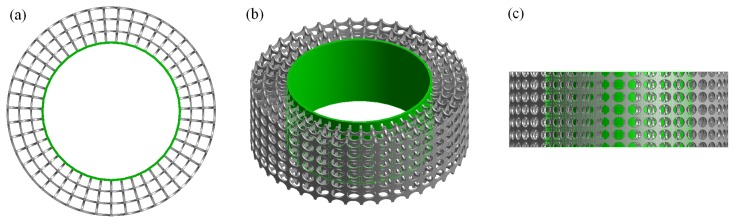
CAD images of the SPCC revealing the LOS in the (**a**) spanwise direction and (**b**,**c**) radial direction. The inner cylinder is illuminated in a green shading to help visualize the radial LOS in images (**b**,**c**). The images are rotated about the horizontal axis (i.e., the flow direction) from (**a**) to (**c**). The CAD model and images are created using ANSYS Workbench Geometry.

The uniform pore structure ensures a clear LOS from the bare cylinder surface to the outer layer of the porous cover. Furthermore, there also exists a clear LOS along the spanwise direction. These LOSs will be useful for future flow measurements, including flow visualization tests (spanwise direction: smoke visualization or PIV of the flow field within the pores) and hot-wire anemometry tests (radial direction: inserting a probe into the porous structure and measuring the velocity profile within the porous media). These LOSs are illustrated in Figure 4.

To evaluate the PPI of the SPCC requires some assumptions and basic calculations. The size of the pores of the SPCC on the surface are elliptical, with the long-axis distance of 3 mm and a short-axis distance of 2.2 mm (corresponding to 2r). Due to the linear scaling of each porous layer within the coating in the radial direction only, the pore dimensions decrease closer to the cylinder center, where the layer closest to the cylinder center are circular with a diameter of 2.2 mm. Thus, across four porous layers the mean long-axis pore distance is 2.6 mm. In the radial direction, the average distance between the ellipse-shaped pores is 2.9 mm. Therefore the average pore dimension is the average of long-axis mean = 2.6 mm, short-axis = 2.2 mm and radial-axis mean = 2.9 mm, which is calculated to be 2.57 mm. Thus, the SPCC has PPI = 25.4 mm/2.57 mm ≈ 10, closely matching the PPI of the PUPCC and MFPCC. The porosity, ϕ (%), of the PCCs were calculated using the following relationship

(1) $$\phi =100\times \left(1-\frac{V_{\mathrm{PCC}}}{V_{S}}\right)$$

where $V_{\mathrm{PCC}}$ is the volume of the PCC and $V_{S}$ is the volume of a solid annulus with inner diameter *d* and outer diameter *D*. For the SPCC, ANSYS Workbench Geometry can simply provide $V_{\mathrm{PCC}}$. For the MFPCC and PUPCC, each of the cylinders are weighed and knowing their material density, $V_{\mathrm{PCC}}$ is then calculated and thus divided by the same $V_{S}$ as used in the SPCC calculation. Each of the PCCs possess 10 PPI. The porosities vary slightly, where the MFPCC, PUPCC and SPCC have a porosity value of ϕ = 90%, 95% and 87%, respectively.

### 2.4. Other Manufactured Examples

**Figure 5:**
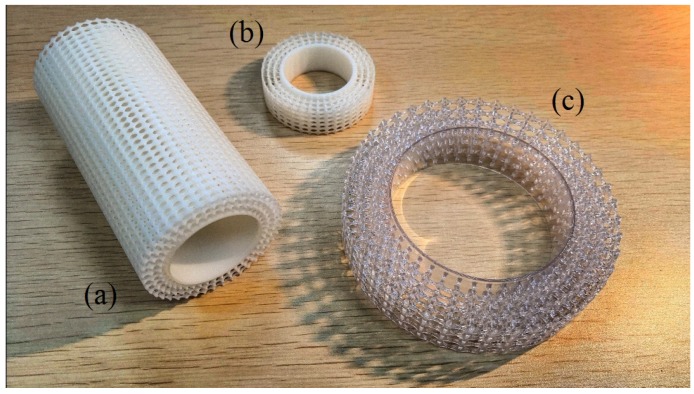
Some SPCC 3-D printed samples. Each of the cylinders use the same design as presented in this paper, yet are scaled in the radial direction to produce cylinders of different *D* but with t/d=0.25. (**a**) *d* = 40 mm, *D* = 60 mm, (**b**) *d* = 32 mm, *D* = 48 mm and (**c**) *d* = 64 mm, *D* = 96 mm.

Figure 5 presents some 3-D printed SPCC samples that possess the same c-shape parameters as presented in Figure 3. The simplicity of the design allows the model to be globally expanded in the radial direction only, such that the internal porous structure retains the same proportions. This is achieved by expanding the blueprint structure, as presented in Figure 3f. An exact 3-D printed copy of Figure 3 is presented in Figure 5a. The small white sample (Figure 5b) possesses *D* = 48 mm as a trial experiment, yet also retains the same internal structure proportions via the use of a radial expansion of the blueprint. The largest sample (Figure 5c) has *D* = 96 mm and is highly transparent, as it is 3-D printed using a transparent ultraviolet curing epoxy resin. This could allow the use of Index Refraction Matching within a water tunnel facility [22] as discussed in Section 1, or tomographic PIV [20] to investigate the internal flow field of a PCC. To 3-D print a customized randomized porous structure, let alone a highly transparent one, would present a significant challenge.

This design while seemingly complex has much flexibility. Each of the parameters in Figure 3a can be varied to achieve a required porosity and/or PPI. The outer diameter, *D*, can be linearly scaled to produce a different cylinder diameter with the same porosity and maintaining the internal porous structure. The pore size can also be easily controlled for small or larger cylinder diameters; this unfortunately is hard to achieve using metal foams or porous polyurethane due to the limited range of PPIs available from suppliers. This structured design also allows the customization of porous structures for future optimization of flow and noise control (such as non-uniform porosity gradient around the cylinder circumference and span).

### 2.5. Experimental Method

**Figure 6:**
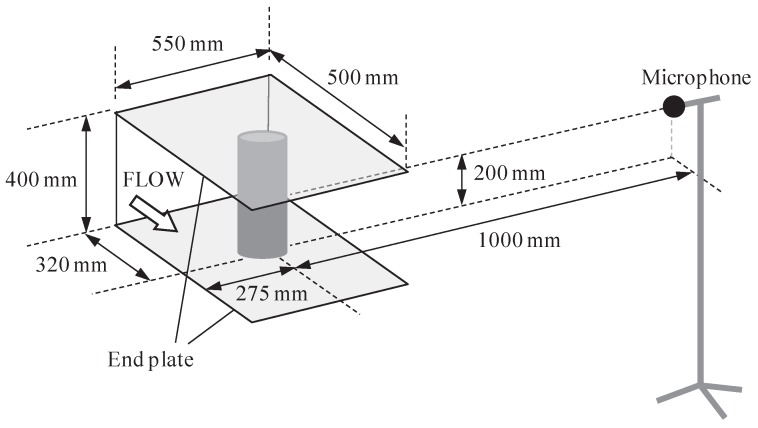
Schematic diagram representing the testing configuration in the 550 mm × 400 mm aeroacoustic wind tunnel in CARDC.

Acoustic tests were conducted at the Key Laboratory of Aerodynamic Noise Control, China Aerodynamics Research and Development Center (CARDC) in Mianyang, China. The closed-circuit aeroacoustic wind tunnel used in this study has a test section area of 550 mm (wide) × 400 mm (high). Taking into account the acoustic wedges attached to the walls, the wind tunnel chamber dimensions are approximately 3 m (wide) × 4 m (long) × 3 m (high). The turbulence intensity is ≤0.05% when using an open test section configuration and the blockage ratio using the PCCs is less than 11%. A schematic diagram representing the testing configuration is presented in Figure 6, identifying the test section, cylinder and microphone location. The testing conditions are listed in Table 1. Each of the cylinders were subject to the same flow speeds, yet it should be noted that the Reynolds numbers are calculated based on the cylinder outer diameter and thus the bare cylinder Reynolds numbers are less than the PCCs.

Acoustic data was collected using a single Brüel & Kjær (B&K, Copenhagen, Denmark) 1/2" free-field microphone positioned in the acoustic far field to determine the Power Spectral Density (PSD) of the self-noise of the cylinders placed in uniform flow. Measurements were obtained at a sampling rate of $2^{15}$ = 32,678 Hz over a sampling period of 16 s which were then passed through high and low pass filters set at 30 Hz and 20,000 Hz, respectively. The MATLAB (The MathWorks, Inc.) pwelch function was used to produce estimates of the PSD, by segmenting the data into eight equal lengths that were windowed using a Hanning window of equal length. The PSD (Pa/Hz) results are presented with 1 Hz resolution. The background data were obtained with the sideplates that support the cylinder in place for a range of flow speeds, from 20 m/s to 50 m/s.

The cylinders were attached to the test contraction side plates using smooth metallic fittings that protrude inside the PCC and into the side plate. The section of the fittings that were exposed to the flow have a 20 mm exposed length and a 60 mm diameter, thus ensuring a flush mating with the PCC to minimize any potential interference noise. Due to the development of the boundary layer at along the side plates, negligible bare-cylinder-related vortex shedding behavior is expected at the cylinder ends. In addition, the comparison between the SPCC and other PCCs is unaffected as the fittings are the same for each PCC type. Please note that the 360 mm PCC cylinder spans combined with 2 × 20 mm fitting cylindrical lengths match the test section height of 400 mm.

### 2.6. Numerical Method

A basic Computational Fluid Dynamics (CFD) analysis was conducted to compare the difference in wake structures and boundary layer development of the SPCC and a typical randomized porous structure, as approximated using a Darcy–Forchheimer Model (DFM). The OpenFOAM (OpenCFD Ltd., ESI Group) open-source software code (pimpleFoam solver) was used to solve the transient flow analyses of two types of porous cylinders. The inlet flow speed is *U* = 10 m/s for both cylinder cases. The cylinder dimensions are identical to the experimental conditions, such that *t* = 10 mm, *d* = 40 mm and *D* = 60 mm. To reduce the computational cost of the SPCC simulation, a quasi-3D model was used that was six elements thick. The computational domain side surfaces are symmetry boundaries and the top and bottom surfaces are far-field boundaries. Solutions were obtained in $10^{-5}$ second time-step intervals over a total time of 10 s. To model the flow field within the SPCC model, a standard k−ϵ turbulence model was used within a computational domain divided into $2.5\times 10^{6}$ cells, primarily concentrated within the porous layer, whereas the DFM requires far fewer grid cells ($4\times 10^{5}$). The DFM requires the use of the semi-empirical Ergun porous model [28] to obtain necessary DFM parameters [29,30,31] that match the PPI and porosity parameters of the SPCC described in Section 2.1.

## 3. Results

The intention of these results is to show the similarity in recorded acoustic measurements and flow behavior between the SPCC and other PCC types, thereby validating the SPCC as an alternative to common porous materials for passive flow and noise control.

### 3.1. Experimental Validation

**Figure 7:**
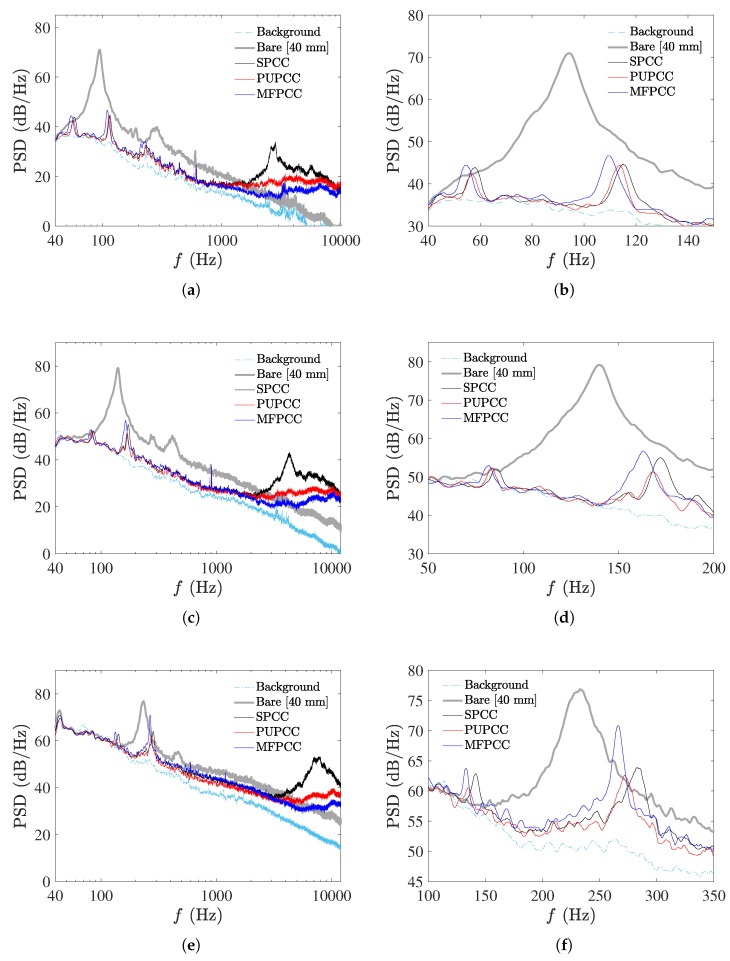
PSD of the PCC’s (**left**) and magnification within the tonal noise frequency range (**right**) at (**a**,**b**) *U* = 20 m/s, (**c**,**d**) *U* = 30 m/s and (**e**,**f**) *U* = 50 m/s.

The measured acoustic PSDs of the *d* = 40 mm bare cylinder and the *D* = 60 mm PCC’s are presented in Figure 7 for flow speeds *U* = 20 m/s, 30 m/s and 50 m/s. On the right-side of the figures, enlargements of the figures on the left are presented to help see the difference in tone magnitudes and frequencies.

**Figure 8:**
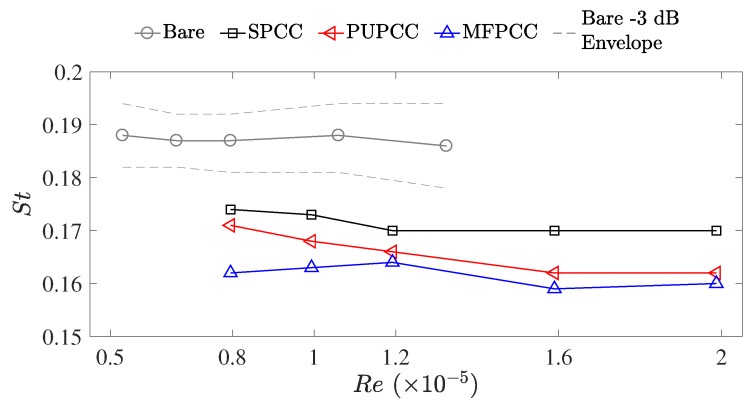
Strouhal numbers, St, of the bare cylinder shedding tone, $f_{\mathrm{BARE}}$, and the lower-frequency tone of the PCCs, $f_{\mathrm{PCC}}$. The dashed line represents the bare cylinder −3 dB bandwidth window that very closely matches published data [16].

It can be easily observed that applying the porous coatings causes significant reduction of the vortex shedding tone produced by the bare cylinder for all flow speeds presented. Furthermore, each of the PCCs display two tones which is consistent with Geyer et al. [8] who investigated various types of PCCs at similar Reynolds number conditions. The differences in tonal magnitudes and frequencies between each of the PCCs are not significant and may be due to the slight variations in porosity. This reveals that the SPCC has similar tonal noise reduction properties as the other PCCs and therefore reveals a similar (or equally effective) vortex shedding reduction characteristic. The lower-frequency tone appears to correspond to the vortex shedding tone of the outer diameter, *D* = 60 mm, due to its corresponding Strouhal number laying within the range of 0.16 to 0.18 (these Strouhal number data are further discussed and presented in Figure 8). The higher-frequency tone, in all flow cases and for all PCC cylinder types, is twice the lower tone frequency. Typically, such a tone would be recognized as a primary harmonic of the shedding tone; however, its magnitude in most cases exceeds the shedding tone magnitude, especially at higher Reynolds numbers. Consideration was given to any resonances within the test section and other non-anechoic facility effects that may cause strong reflections and thus amplifications of specific frequencies, yet if they were to exist, such strong secondary tone behavior would also be observed with the bare cylinder shedding frequency, yet this data very closely matches theory and extensive previously published data [16].

By observation of Figure 7a, it is observed that at *U* = 20 m/s and f≥1000 Hz each of the PCCs show increased high-frequency noise contributions relative to the bare cylinder. The SPCC has a significant broadband contribution with a peak magnitude of 34 dB focused between *f* = 2600 Hz to 2800 Hz. This trend is observed with increasing flow speed (in Figure 7a,c,e, where the broadband contribution that exceeds the bare cylinder broadband noise increases in frequency and magnitude). It is postulated that the high-frequency contribution of the SPCC is due to a complex 3-D flow interaction of cavity modes on the outer-diameter surface and also within the porous structure that only occurs due to the regularity of the pore structures. To validate this postulation, a hot-wire anemometry probe placed near the outer cylinder surface and within the pores, while simultaneously obtaining acoustic data in the far field, can be used to detect any high-frequency fluctuations in these regions and the coherence between the velocity and acoustic signals.

As described in Section 2.3 and depicted in Figure 3g, the SPCC blueprint is cloned along the spanwise axis to produce a SPCC of a desired span. The distance between each cloned SPCC blueprint is equal (i.e., equispaced). To avoid some of the cavity interaction that potentially leads to the higher-frequency noise contributions shown in Figure 7, the distance between each cloned SPCC blueprint can be slightly modified (i.e., non-equispaced) to vary the porosity and PPI in the spanwise direction. This would in turn would affect the spanwise coherence of the SPCC and potentially break-up some of the acoustic interactions that lead to the higher-frequency broadband contributions. While this is currently a proposition, it must be noted that the SPCC design presented here is an initial design and it is not optimized for passive flow control. It is, however, able to be modified and adapted for improved passive noise control for specific flow regimes whereas typical randomized porous media are not.

To investigate any effects of the facility on the bare cylinder shedding tone, the frequency range of the −3 dB bandwidth was measured and plotted as dotted lines in Figure 8. This bandwidth shows a near-exact match with the bandwidth data presented in Norberg et al. [16] which adds further evidence to the measured spectra bearing negligible adverse impact due to the testing facility. The calculated Strouhal numbers of the bare cylinder shedding tones (*d* = 40 mm) and the lower-frequency tones of the PCCs (*D* = 60 mm) are presented in Figure 8. The lower-frequency tones of the PCCs display lower Strouhal numbers than the bare cylinder. The SPCC presents the highest Strouhal numbers of the PCCs, with an average value of St≈0.171. With increasing Reynolds number, the PUPCC and MFPCC present similar Strouhal numbers approaching a value of St≈0.162. The differences of the PCC Strouhal numbers and the bare cylinder Strouhal numbers indicate that the lower-frequency tone, while likely strongly linked to an outer-diameter vortex shedding process, is impeded by the porous structure and results in a unique vortex shedding processes within the wake.

**Figure 9:**
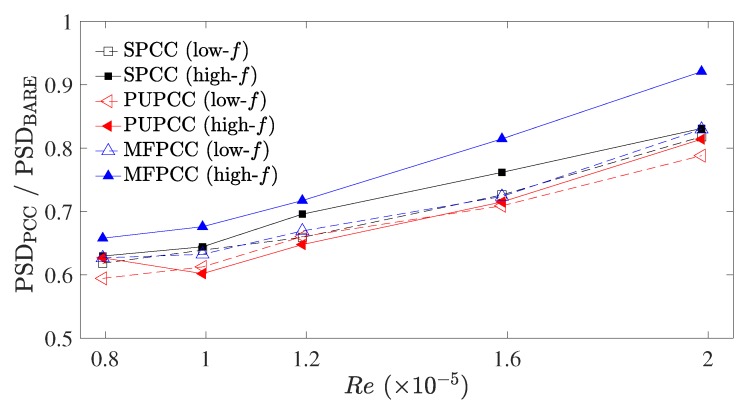
Magnitudes of the lower-frequency and higher-frequency tone magnitudes, ${\mathrm{PSD}}_{\mathrm{PCC}}$, relative to the bare cylinder shedding tone magnitude, ${\mathrm{PSD}}_{\mathrm{BARE}}$.

Figure 9 presents the relative magnitudes of the PCCs, ${\mathrm{PSD}}_{\mathrm{PCC}}$, with respect to the bare cylinder shedding tone, ${\mathrm{PSD}}_{\mathrm{BARE}}$. Both the lower and higher-frequency tones, corresponding to the second and first tones of the acoustic spectra presented in Figure 7, increase in relative magnitude with increasing Reynolds number. The MFPCC displays the highest relative amplitude for the higher-frequency tone and on average, the PUPCC displays the lowest relative magnitudes for both the low and high-frequency tones. From these data, it can be observed that overall the MFPCC produces the greatest tonal noise, yet it appears to suppress the lower-frequency shedding tones as well as the other PCCs. The PUPCC is the quietest cylinder of the three PCCs considered in this study, despite producing slightly greater higher-frequency broadband contributions than the MFPCC, as shown in Figure 7. The SPCC produces similar lower-frequency tone suppression as the other PCCs. This is an important result. This implies that the internal flow field of the SPCC should also reveal similar characteristics as the more commonly used MFPCC and PUPCC. The SPCC can be far more easily customized for different experimental facilities and detailed flow experiments which are otherwise impossible with randomized porous structures. The relative simplicity of the SPCC design, relative to a randomized porous structure, allows the direct simulation of the internal flow field of the porous coating without needing to employ approximations that are otherwise necessary to model porous media.

The most important experimental observation here is that the SPCC possesses similar passive noise control capability as compared to the MFPCC and the PUPCC, albeit some additional high-frequency contributions. Therefore, the mechanisms responsible for reducing the vortex shedding tone of the bare cylinder are likely to be the same or very similar for each of the PCC types.

### 3.2. Numerical Validation

**Figure 10:**
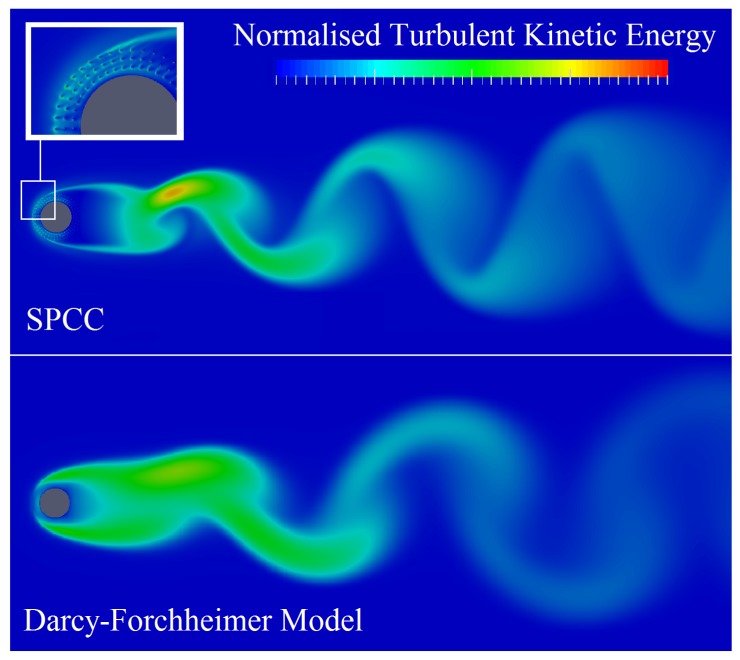
Preliminary CFD result comparing the normalized turbulent kinetic energy distributions of the SPCC with a cylinder using a Darcy–Forchheimer model. Both cylinder model data are normalized to the same scale. Flow is from left to right.

Normalized turbulent kinetic energy plots of the SPCC and a DFM cylinder are presented in Figure 10, displaying an instantaneous snapshot at a solution time of approximately 2 s. Figure 10 illustrates the similarities of the turbulent kinetic energy flow structures between the SPCC and the DFM cylinder and that the von-Karman street is very similar for both cylinder models. The wake structure, from the cylinder lee-surface to approximately four cylinder diameters downstream, shows a predominately turbulent flow region for both cylinders with the SPCC showing a greater local maximum. The boundary layer development on the leading surface of the DFM cylinder is thicker than the SPCC. Overall, the two CFD results bear significant flow similarities and most importantly, the flow behavior of the SPCC appears very similar to the DFM flow that represents typical randomized porous media.

**Figure 11:**
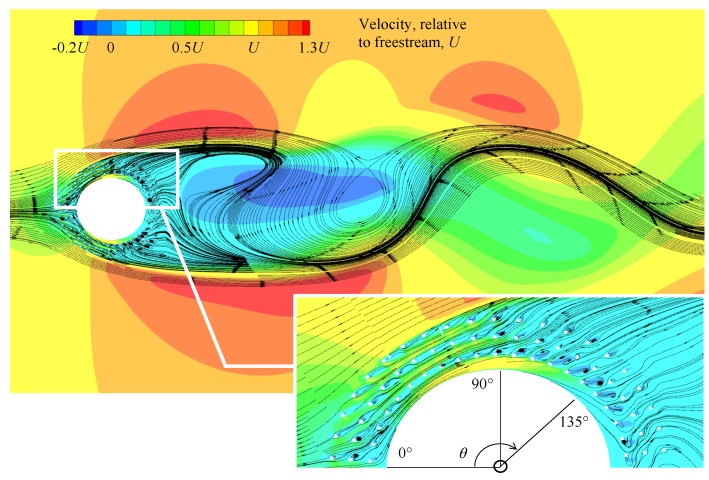
Preliminary CFD result of the streamlines and velocity contours of the SPCC, revealing the flow field inside the structured porous media and the near-wake. Circumferential locations, θ, are identified to assist in-text explanations. Flow is from left to right.

To further illustrate the flow field for the same flow and simulation conditions, Figure 11 presents the velocity contours and streamlines of the SPCC inside the porous layer and near-wake. From the circumferential angle $\theta =0^{\circ }$ to 90${}^{\circ }$, it can be observed that the streamlines follow the internal path of the porous medium. Each solid component of the porous medium (that can be identified as small white squares in Figure 11) generates a small wake, resulting in the production of turbulent kinetic energy within each pore. From $\theta =90^{\circ }$ to 135${}^{\circ }$, the flow no longer remains within the circular paths of the porous medium and travels between adjacent pores. Aft of each solid component, small regions of reverse flow are observed with complex flow structures. From $\theta =90^{\circ }$ to the rear of the cylinder, the streamlines converge downstream thus generating a typical broad cylinder wake, with a region of significant reverse flow. Further in the wake, the streamlines represent typical vortex shedding behavior that is expected behind a bare cylinder in this Reynolds number range [3,16,32].

By investigation of these numerical results it can be seen that the SPCC provides a visualization of the internal flow field that is not possible using the DFM or similar alternatives. The results presented in Figure 10 and Figure 11 reveal very fine details of the flow around each pore and the complex flow interaction of the flow due to the influence of each pore on the flow field. Furthermore, the near and far wake regions possess similarity, thereby showing that the internal and external flow fields of the SPCC and randomized PCC (approximated by the DFM) are also similar. Further numerical simulations for the SPCC are in progress which may help shed more light on the complex 3-D flow interaction within the porous medium and also cavity modes that are suspected from the acoustic results presented in Figure 7.

## 4. Conclusions

The design and validation of a novel yet customizable structured porous coated cylinder are presented. The structured porous coated cylinder design is easily adaptable to achieve various PPI and porosity specifications via 3-D printing. The acoustic measurements of a structured porous coated cylinder are compared with well-documented metal foam and polyurethane porous coated cylinders, each of which display significant noise reduction relative to the bare cylinder, implying that the structured porous coated cylinder possesses similar (or the same) passive noise control properties as a randomized-structure porous coated cylinder. This important result leads to the ability to analyze the much simpler structured porous material geometry, using both numerical methods and experimental fluid mechanics to understand the flow behavior within the porous medium that causes the reduction of the cylinder vortex shedding tone. Some numerical results are also presented that provide an insight to the highly complex flow field within a porous medium and a qualitative comparison of the structured porous flow field with that of a Darcy–Forchheimer model, representing typical randomized porous media. Agreement between the structured and approximated-randomized porous structure is observed in the near and far wake.

Future work will involve designing a range of structured porous coated cylinders that possess non-uniform PPI around the cylinder circumference, as a means of optimizing the passive flow and noise control of a bare cylinder. This will also allow deeper research into the fundamental fluid mechanics responsible for the vortex shedding tone reduction within the structured porous coating layer, both numerically and experimentally (e.g., via the use of tomographic PIV and hot-wire anemometry measurement methods).

## Figures and Tables

**Table 1:** Experimental flow conditions for the bare and PCCs.

Cylinder Type	Testing Conditions			
PCCs	*U* (m/s)	20	30	50
(*D* = 60 mm)	$Re_{D}\;\left(\times 10^{-5}\right)$	0.8	1.2	2.0
Bare	*U* (m/s)	20	30	50
(*d* = 40 mm)	$Re_{d}\;\left(\times 10^{-5}\right)$	0.5	0.8	1.3

## Nomenclature

*d*	[mm]	bare cylinder diameter
*D*	[mm]	porous coated cylinder outer diameter
*f*	[Hz]	acoustic frequency
*h*	[mm]	height of c-shaped chip
*l*	[mm]	thickness of c-shaped chip
*L*	[mm]	cylinder span
*r*	[mm]	pore radius of c-shaped chip
Re	[-]	Reynolds number
$Re_{\mathrm{av},\min}$	[-]	Minimum-average Reynolds number
$Re_{\mathrm{av},\max}$	[-]	Maximum-average Reynolds number
St	[-]	Strouhal number
*t*	[mm]	porous coating thickness
*U*	[m/s]	freestream flow velocity
$V_{\mathrm{PCC}}$	[$m^{3}$]	porous coated cylinder volume
$V_{S}$	[$m^{3}$]	equivalent solid cylinder volume
*w*	[mm]	width of c-shaped chip
ϕ	[%]	porosity
θ	[${}^{\circ }$]	circumferential angle

## Abbreviations

The following abbreviations are used in this manuscript:

B&K	Brüel & Kjær
CAD	Computer Aided Design
CARDC	China Aerodynamics Research and Development Center
CFD	Computational Fluid Dynamics
DFM	Darcy–Forchheimer Model
LOS	Line-Of-Sight
MFPCC	Metal Foam Porous Coated Cylinder
PCC	Porous Coated Cylinder
PPI	Pores Per Inch
PSD	Power Spectral Density
PUPCC	Polyurethane Porous Coated Cylinder
SPCC	Structured Porous Coated Cylinder

## References

[1] Boorsma K., Zhang X., Molin N., Chow L.C. (2009). Bluff Body Noise Control Using Perforated Fairings. AIAA J..

[2] Sueki T., Ikeda M., Takaishi T. (2009). Aerodynamic noise reduction using porous materials and their application to high-speed pantographs. Q. Rep. RTRI.

[3] Zdravkovich M. (1981). Review and classification of various aerodynamic and hydrodynamic means for suppressing vortex shedding. J. Wind Eng. Ind. Aerodyn..

[4] Sueki T., Takaishi T., Ikeda M., Arai N. (2010). Application of porous material to reduce aerodynamic sound from bluff bodies. Fluid Dyn. Res..

[5] Aguiar J., Yao H., Liu Y. Passive Flow/Noise Control of a Cylinder Using Metal Foam. Proceedings of the 23rd International Congress on Sound and Vibration.

[6] Ruck B., Klausmann K., Wacker T. (2011). The flow around circular cylinders partially coated with porous media. AIP Conf. Proc..

[7] Klausmann K., Ruck B. (2017). Drag reduction of circular cylinders by porous coating on the leeward side. J. Fluid Mech..

[8] Geyer T.F., Sarradj E., Herold G. Flow Noise Generation of Cylinders with Soft Porous Cover. Proceedings of the 21st AIAA/CEAS Aeroacoustics Conference.

[9] Geyer T.F., Sarradj E. (2016). Circular cylinders with soft porous cover for flow noise reduction. Exp. Fluids.

[10] Geyer T.F., Sharma S., Sarradj E. Detached Eddy Simulation of the Flow Noise Generation of Cylinders with Porous Cover. Proceedings of the 2018 AIAA/CEAS Aeroacoustics Conference.

[11] Liu H., Azarpeyvand M., Wei J., Qu Z. (2015). Tandem cylinder aerodynamic sound control using porous coating. J. Sound Vib..

[12] Liu H., Azarpeyvand M. Passive Control of Tandem Cylinders Flow and Noise Using Porous Coating. Proceedings of the 22nd AIAA/CEAS Aeroacoustics Conference.

[13] Liu H., Wei J., Qu Z. (2012). Prediction of aerodynamic noise reduction by using open-cell metal foam. J. Sound Vib..

[14] Arcondoulis E., Liu Y. The Effect of Porosity on the Porous Coated Cylinder Diameter. Proceedings of the Australian Acoustical Society AAS2018 Adelaide.

[15] Szepessy S. (1994). On the spanwise correlation of vortex shedding from a circular cylinder at high subcritical Reynolds number. Phys. Fluids.

[16] Norberg C. (2003). Fluctuating lift on a circular cylinder: Review and new measurements. J. Fluids Struct..

[17] Bruneau C.H., Mortazavi I. (2008). Numerical modelling and passive flow control using porous media. Comput. Fluids.

[18] Xu C., Mao Y., Hu Z. (2018). Numerical study of pore-scale flow and noise of an open cell metal foam. Aerosp. Sci. Technol..

[19] Naito H., Fukagata K. (2012). Numerical simulation of flow around a circular cylinder having porous surface. Phys. Fluids.

[20] Arcondoulis E., Ragni D., Rubio Carpio A., Avallone F., Liu Y., Yang Y., Li Z. The Internal and External Flow Fields of a Structured Porous Coated Cylinder and Implications on Flow-Induced Noise. Proceedings of the 25th AIAA/CEAS Aeroacoustics Conference.

[21] Triantafyllou G.S., Triantafyllou M.S., Chryssostomidis C. (1986). On the formation of vortex streets behind stationary cylinders. J. Fluid Mech..

[22] Bai K., Katz J. (2014). On the refractive index of sodium iodide solutions for index matching in PIV. Exp. Fluids.

[23] Geyer T.F., Kamps L., Sarradj E., Brücker C. Passive Control of the Vortex Shedding Noise of a Cylinder at Low Reynolds Numbers Using Flexible Flaps. Proceedings of the 23rd AIAA/CEAS Aeroacoustics Conference.

[24] Yuan H., Xia C., Chen Y., Yang Z. Flow around a Finite Circular Cylinder Coated with Porous Media. Proceedings of the 8th International Colloquium on Bluff Body Aerodynamics and Applications.

[25] Jenkins L., Neuhart D., McGinley C., Khorrami M., Choudhari M. Measurements of Unsteady Wake Interference between Tandem Cylinders. Proceedings of the 36th AIAA Fluid Dynamics Conference and Exhibit.

[26] Neuhart D., Jenkins L., Choudhari M., Khorrami M. Measurements of the Flowfield Interaction between Tandem Cylinders. Proceedings of the 15th AIAA/CEAS Aeroacoustics Conference.

[27] Hutcheson F.V., Brooks T.F., Lockard D.P., Choudhari M.M., Stead D.J. Acoustics and Surface Pressure Measurements from Tandem Cylinder Configurations. Proceedings of the 20th AIAA/CEAS Aeroacoustics Conference.

[28] Ergun S. (1952). Fluid flow through packed columns. Chem. Eng. Prog..

[29] Vafai K. (1984). Convective flow and heat transfer in variable-porosity media. J. Fluid Mech..

[30] Hsu C., Cheng P. (1990). Thermal dispersion in a porous medium. Int. J. Heat Mass Transf..

[31] Alazmi B., Vafai K. (2000). Analysis of variants within the porous media transport models. J. Heat Transf..

[32] Chen W.L., Gao D.L., Yuan W.Y., Li H., Hu H. (2015). Passive jet control of flow around a circular cylinder. Exp. Fluids.

